# Acute-Stage Mental Health Symptoms by Natural Disaster Type: Consultations of Disaster Psychiatric Assistance Teams (DPATs) in Japan

**DOI:** 10.3390/ijerph182312409

**Published:** 2021-11-25

**Authors:** Yoshifumi Takagi, Sho Takahashi, Yasuhisa Fukuo, Tetsuaki Arai, Hirokazu Tachikawa

**Affiliations:** 1 Faculty of Humanities, Tokyo Kasei University, Tokyo 173-8602, Japan; cch61030@gmail.com; 2 Department of Disaster and Community Psychiatry, Faculty of Medicine, University of Tsukuba, Tsukuba 305-8575, Japan; tachikawa@md.tsukuba.ac.jp; 3 Ibaraki Prefectural Medical Center of Psychiatry, Kasama 309-1717, Japan; 4 Disaster Psychiatric Assistance Teams (DPATs) Secretariat, Commissioned by the Ministry of Health, Labor and Welfare, Tokyo 108-8554, Japan; josukef3@gmail.com; 5 Shinkeikahamamatsu Hospital, Hamamatsu 432-8013, Japan; 6 Department of Psychiatry, Division of Clinical Medicine, Faculty of Medicine, University of Tsukuba, Tsukuba 305-8575, Japan; 4632tetsu@md.tsukuba.ac.jp

**Keywords:** disaster, volcanic eruption, landslide, flood, and earthquake, DMHISS, disaster psychiatry, Japan, acute mental health symptoms, DPAT (Disaster Psychiatric Assistance Team)

## Abstract

This study analyzed the support activities that the Disaster Psychiatric Assistance Team (DPAT) in Japan provided following four previous disasters (a volcanic eruption, a mudslide, a flood, and an earthquake) to identify links between the disaster type and the characteristics of acute stage mental disorders observed. Using Disaster Mental Health Information Support System database records of consultations with patients supported by the DPAT during the survey period from 2013 (when DPAT was launched) to 2016, we performed cross-tabulations and investigated significant differences using chi-squared tests. For expected values less than 5, Fisher’s exact test was performed. Frequently occurring acute-stage symptoms after a disaster include anxiety, sleep problems, mood and affect, and physical symptoms. The affected population characteristics, victim attributes, severity of damage sustained, and evacuation status were the chief factors that influenced acute-stage mental health symptoms. The psychiatric symptoms detected in our study together with the results of diagnoses are important for determining the types of early interventions needed during the acute stage of a disaster. By sharing baseline mental health information, together with disaster-related characteristics highlighted in this study, mental health providers are better able to predict future possible mental disorders and symptoms.

## 1. Introduction

Natural disasters refer to the results of natural phenomena such as earthquakes, floods, fires, volcanic eruptions, tsunamis, and droughts [1]. In addition to causing human suffering, such as physical injuries, natural disasters have far-reaching impacts on the lives of disaster victims, including loss of home, property, and jobs, difficulties in securing food, and disruptions to transportation and lifelines. Furthermore, post-disaster victims are forced to live a different life from their pre-disaster life, and various mental health problems, such as stress reactions and maladjustment to the new lifestyle, become apparent. Norris et al. summarized the studies on 225 natural disasters reported between 1981 and 2004 and reported several stressors affecting the mental health of disaster victims, including gender, loss experiences, life-threatening experiences, panic during the disaster, financial loss, displacement, and a history of mental illness [2]. In addition, the prevalence of mental disorders following a disaster is said to be twice as high as under normal conditions in the affected areas [3]. Thus, natural disasters cause both physical and mental disorders in their victims, creating a need for related medical assistance.

A typical mental disease that develops after a disaster is post-traumatic stress disorder (PTSD) [4,5]. It is evident from past studies that the incidence of PTSD and depression increases after a disaster [6,7]. Another report that reviewed studies from 1982 to 2017 and focused on PTSD and depression indicated that depressive symptoms increase as social capital decreases [8]. Although many mental diseases caused by disasters are perceived to be temporary, and improve after several days or weeks [4], many researchers state that 30–40% of PTSD cases in the wake of natural disasters take a chronic course [9], with mental health problems becoming prolonged [10,11,12,13]. In a report published following Hurricane Katrina, researchers investigated K6 and IES-R scores twice: 7–19 months and 43–59 months after the hurricane, and found a high percentage of the victims to be still suffering post-traumatic stress symptoms (PTSS) [9]. Moreover, a survey conducted twelve years after the same hurricane disaster reportedly found symptoms that manifested as PTSD in one of six victims [10]. In a study published after the Great Hanshin-Awaji Earthquake that investigated subjects who had been hospitalized during a three-month period after the disaster, a large number of existing schizophrenia patients whose houses had been damaged were reported to have experienced a worsening of hallucinations, delusions, and other symptoms [14]. A qualitative survey, conducted twenty years after this earthquake, found emotions such as sorrow, fear, and remorse still lingering among the survivors as memories of the earthquake [13].

However, most of these reports are epidemiological studies of mental disorders that emerged in the mid- to long-term in relation to a specific disaster. Few statistical studies have been conducted, and what mental health symptoms are characteristic of the acute phase of a disaster and whether they are influenced by the type of disaster or the disaster situation is unclear. [15]. One possible reason for this gap in the literature was the lack of a psychiatric team providing support during the acute phase, making it difficult to assess the affected people, including their mental health needs in the acute phase.

In Japan, since the Great East Japan Earthquake that occurred in 2011, the Disaster Psychiatric Assistance Team (DPAT) has been providing mental health medical assistance as a highly-organized group that specializes in acute-stage psychiatric medical care [16]. DPAT conducts outreach activities to provide mental health care to disaster victims and supporters in the affected areas within 48 h after the disaster. The main activities are (1) providing psychological support to evacuees at evacuation centers, (2) transporting patients from affected psychiatric hospitals, and (3) supporting the supporters in the affected areas [16,17]. The DPAT is organized by a multi-professional team that includes psychiatrists, nurses, and logisticians such as social workers and psychologists. Not only evacuees who have been consulted by DPAT, but all evacuees are targeted, and psychiatrists take the lead in assessing symptoms and providing specialized support.

The DPAT makes use of the Disaster Mental Health Information Support System (DMHISS) and records their support activities in the form of digital data [16], rendering a statistical investigation possible. The DMHISS was developed in FY2012 by Toshiba Digital Solutions Corporation on consignment from the National Information Center of Stress and Disaster Mental Health, to integrate the activity records of the mental health and medical care support teams that engage in operations during disasters, and ensure that their assistance meets the support needs of the disaster-stricken areas. It began operating nationwide in March 2013 as an internet-based information system. The functions of the DMHISS are divided into four stages: normal times (prior registration of the support teams); the initial response (organizing the dispatch of the support teams); during activity (gathering of activity records); and after activity (compilation and analysis of activity records). There are two types of tools for gathering activity records: individual data for compiling consultation records of interviewees whom DPAT had dealt with individually; and reports that record each DPAT team’s daily activities. The individual data are composed of basic information such as the names of the team and its members who oversaw the consultation, and the course of the consultations. Attributes, such as the interviewees’ attributes (gender, age, disaster status, died/missing; injury to the interviewee himself/herself; injury to family members, relatives, and/or acquaintances; collapse of house; forced evacuation from home; loss of property other than the house, etc.); background to the consultation (loss of close relatives, changes in housing environment, problems with financial and livelihood reconstruction, loss of jobs and employment problems, interpersonal relationships, family problems, education, childrearing, change of school, health problems); medical history, time of onset (before/after the disaster); symptoms at the time of medical examination/treatment, diagnosis; and content of responses, are included [16,18,19].

Some reports review DPAT’s support activities provided during past disasters, which verify the duration of their activities and other matters [16,17]. However, there are no reports that investigate the mental and medical needs and the characteristics of mental disorders seen during the acute stage in a way that is specific to the type of natural disaster such as earthquakes or floods. If we can differentiate the mental health symptoms and characteristics of people that need support in the acute phase of a disaster based on its scale and situation, we can allocate resources appropriately during the acute phase of a disaster mental health response, improve response skills, and prepare for future natural disasters.

Therefore, we aimed to analyze the data on the support activities that the DPAT had provided after four past disasters (a volcanic eruption, a mudslide, a flood, and an earthquake) to identify any links between the type of disaster and the characteristics of mental disorders and mental symptoms seen during the acute stage of the disaster.

## 2. Methods

### Statistical Analysis

Individual data on the four disasters were extracted from the DMHISS database, and datasets comprising individual data were collated. The following survey items were analyzed: Attributes (gender, age, disaster status), Presence or absence of past illness diagnosed, Diagnosis (dementia, alcoholism, schizophrenia, depression, manic depression, acute stress disorder, posttraumatic stress disorder, natural disaster phobia, adjustment disorder, other severe stress reactions, other anxiety disorders, somatoform disorders, sleep disorders, intellectual disability, autistic spectrum disorders, other mental disorders during childhood, epilepsy), Symptoms (physical symptoms, sleep problems, anxiety symptoms, symptoms related to mood and affect, dissociation and conversion symptoms, oppressive symptoms, hallucinations and delusions, behavioral problems, epileptic and convulsive seizures, problems with drinking, disturbance of consciousness, and symptoms unique to children).

The period one month after the disaster was designated the acute stage [17] and, for each of the four disasters, we compared the survey items Gender, Age, Past illnesses, and Symptoms before grouping the four disasters into Diagnosis and Symptoms categories in terms of Gender, Age, Past illnesses, and Disaster status. Symptoms, which summarized the four disasters, were compared with the history of mental disorders. Of the mental disorders, schizophrenia, bipolar disorder, and depression were compared with symptoms. Note that the diagnosis category is a mixture of pre- and post-disaster diagnoses. We performed cross-tabulation and investigated significant differences using the chi-square test. Where the expected value of the cross-tabulation was less than 5, Fisher’s exact test was performed. Measurements were then made using the following criteria: effect size Φ (0.5, and above: strongly related; 0.3–0.49: related; 0.1–0.29: weakly related; below 0.1: unrelated) [20], and Cramer’s V (0.4 and above: Strongly related; 0.25–0.39: Related; 0.1–0.24, weakly related; and <0.1: unrelated) [21]. SPSS Statistics ver. 27 (Armonk, NY, USA) was used for statistical analysis, with a significance level of 5%. This study was carried out with the approval of the Japan Psychiatric Hospitals Association Ethics Committee (approval number 161110-01).

## 3. Results

### 3.1. Patient Selection

In this study, the term from 2013 (when DPAT was launched) to 2016 (when the team’s support activities were recorded in the DMHISS database) was used as the survey period. The research patients were selected from the DMHISS database records of consultations (individual data) with victims of four major disasters for whom the DPAT had carried out support activities during this period (the Mt. Ontake volcanic eruption of 2014 (eruption); the Hiroshima landslide of 2014 (landslide); the Kanto-Tohoku flood of 2015 (flood); and the Kumamoto earthquake of 2016 (earthquake). These were records of consultations requested by the victims and their families who had received assistance at evacuation centers and at home, as well as administrative agencies and health and medical care personnel.

### 3.2. Overview of Four Types of Disasters

#### 3.2.1. Hiroshima Landslide

**Figure 1:**
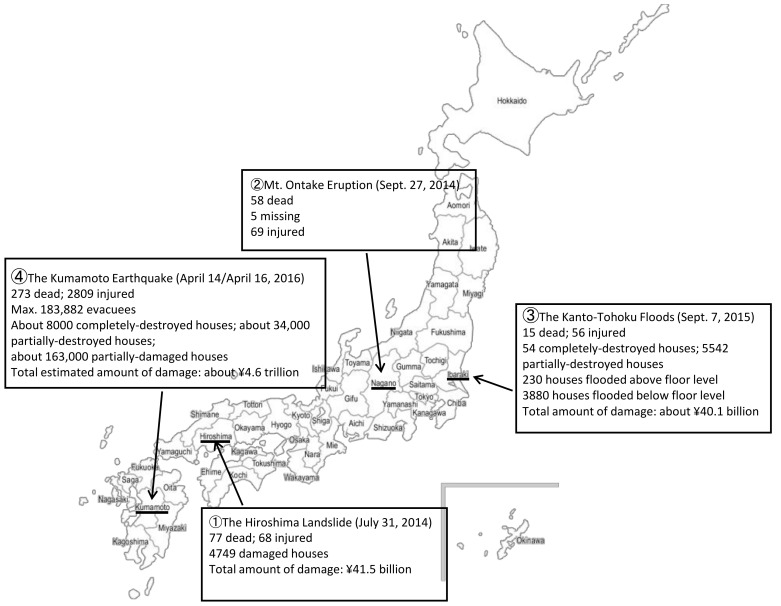
Characteristics of the Four Disasters (Note: Explanation of the date of occurrence, damage and other details of the four disasters).

From 31 July to 11 August 2014, Typhoon Nos. 11 and 12 approached the Japanese archipelago one after the other, causing heavy rain. On 20 August, before dawn, torrential rain fell locally in the northern part of Hiroshima City in the Hiroshima Prefecture, causing large-scale mudslides and landslides at 166 sites. The human toll was 77 deaths and 68 injuries, and 4749 houses were damaged, including 179 destroyed and 217 partially destroyed houses. At the time of the disaster, Hiroshima City had a population of 1,188,067 people. Divided into three cohorts, it consisted of 169,632 people (14.3%) aged 0–14, 743,914 people (62.6%) aged 15–64, and 274,521 people (23.1%) aged 65 and older. The Asaminami Ward, which suffered the heaviest damage, is predominantly a new residential area with the largest population in the city with high birth rates. The Disaster Medical Assistance Team (DMAT) was dispatched to the site immediately. Three Hiroshima DPATs were also dispatched from 22 August to provide psychological support to the evacuation centers for about a month. Thereafter, until the closure of the evacuation center on 25 December, the public health center and other organizations managed the situation. This was the first DPAT dispatch in Japan. [17,22,23] (Figure 1).

#### 3.2.2. The Mt. Ontake Volcanic Eruption

On 27 September 2014, at around 11:52, Mt. Ontake erupted. The mountain is situated along the border between Nagano and Gifu prefectures and is the second-highest volcano in Japan after Mt. Fuji. Ashfall due to the eruption was also observed in Yamanashi Prefecture, 100 km away. The toll of the eruption damage was 58 deaths, 5 missing, and 69 injured. Since the area directly affected was limited to regions close to the summit, residents from the surrounding area suffered no immediate damage and were not subject to any evacuation orders. Many of the victims were mountain climbers from throughout Japan. One Nagano DPAT was dispatched on 28 September, the second overall DPAT dis-patched in Japan. Since some of the hospitals where the victims were transported did not have psychiatrists, DPAT was sent to provide psychological support to the hospitalized victims and their families [17,24,25] (Figure 1).

#### 3.2.3. Kanto-Tohoku Floods

On 7 September 2015, Typhoon No. 18 caused record-breaking heavy rainfall in the Kanto and Tohoku regions. From 9–10 September, the banks of the Kinugawa River in Joso City, Ibaragi Prefecture, broke, causing flooding. In Ibaraki Prefecture, 15 people died, and 56 were injured. The flood swept away homes, resulting in 54 completely destroyed and 5542 partially destroyed houses, with 230 houses flooded above floor level, and 3880 houses flooded below floor level. At the time of the disaster, Joso City had a population of 61,483, divided into three cohorts consisting of 7633 people (12.4%) aged 0–14; 36,451 people (59.3%) aged 15–64 years; and 16,860 people (27.4%) aged 65 and older. The Ibaraki DPAT was dispatched on 13 September. It worked in cooperation with the Japanese Red Cross Society’s psychological care team. We also collaborated with public health nurses in the affected areas to support the evacuation center patrols. Furthermore, we established headquarters with the Japanese Red Cross Society Psychological Care Team in Joso City Hall to provide supporters’ assistance to city hall staff [17,26,27] (Figure 1).

#### 3.2.4. Kumamoto Earthquake

On 14 April 2016, a foreshock of magnitude 6.5 occurred, followed by a magnitude 7 mainshock that struck on 16 April. The series of earthquakes caused 273 deaths, 2809 injuries, and 183,882 evacuees, resulting in approximately 8000 destroyed and 34,000 partially destroyed houses, and an additional 163,000 houses were damaged.

At the time of the disaster, Kumamoto Prefecture had a population of 1,786,170. There were 773,979 evacuees from municipalities with high evacuation rates—Mashiki-town (33,597), Nishihara-village (6802), and Kumamoto City (733,580). Of these, 109,743 (14.2%) were aged 0–14, 476,053 (61.5%) were aged 15–64, and 188,183 (24.3%) were aged 65 and older. This was the first large-scale organizational support program undertaken by DPAT, with a total of 1091 groups dispatched from throughout Japan. DPATs from outside the prefecture, not the disaster area, were dispatched from 15 April to 30 June. They supported hospitals, transported patients, patrolled evacuation centers, and assisted supporters. Immediately following the disaster, we established coordination and base headquarters for activities in the affected areas. As for patient transport, 595 patients were transported to psychiatric hospitals in and outside of the prefecture. Thereafter, transport was transferred to Kumamoto DPAT. On 30 October, all DPAT activities were completed [17,28,29,30] (Figure 1).

### 3.3. Comparison of Basic Attributes, Diagnoses, and Symptoms of the Target Population by Disaster

Of the people in the four disasters 1,806 were in the acute stage—Mt. Ontake eruption (12), Hiroshima landslide (86), Kanto-Tohoku flood (139), and Kumamoto earthquake (1569). Table 1 indicates that women and those in the 20–64 age group accounted for a large proportion. A total of 799 people (44.2%) had a history of mental disease. Diagnostically, schizophrenia had the highest prevalence (21.0%), followed by severe stress reactions (13.5%), acute stress disorder (10.9%), adjustment disorder (10.7%), and dementia (9.2%). Anxiety symptoms had the highest prevalence (26.3%), followed by sleep problems (23.5%), physical symptoms (19.3%), and symptoms related to mood and affect (16.5%).

An examination categorized by type of disaster and gender revealed that more women sought help after the earthquake, whereas more men sought help after the flood. After dividing age into three cohorts (aged below 20, ages 20–64, and 65 and older), we found that, while people seeking assistance after the landslide were predominantly under 20 years of age, a significantly higher percentage of people seeking assistance after the flood were 65 or older. The percentage of people with a history of mental disease was significantly higher in the flood.

The results of the diagnoses showed that acute stress disorder occurred most frequently in the victims of the eruption and landslide, adjustment disorder occurred the most frequently after the flood, and other severe stress reactions occurred the most frequently after the earthquake.

The following occurred significantly more frequently: Anxiety symptoms in victims of the eruption; with the landslide, sleep problems, symptoms related to mood and affect, and symptoms unique to children; and with the flood, physical symptoms, sleep problems, and behavioral problems. No significant differences were observed between the earthquake and various symptoms.

### 3.4. Diagnosis and Symptoms of Four Disasters, and Comparison with the Subjects’ Basic Attributes

In Table 2, we compared the differences in the basic attributes vis-à-vis the diagnoses and symptoms of the four disasters combined. 

As a result of comparing gender and diagnosis, we found that a larger percentage of people diagnosed with alcoholism, schizophrenia, and autism spectrum disorder were men. In contrast, a large percentage of those diagnosed with acute stress disorder, other severe stress reactions, and sleep disorders were women. Moreover, a comparison between gender and symptoms found that men had significantly more behavioral problems, drinking problems, and symptoms unique to children, whereas women had significantly more sleep problems, anxiety symptoms, and dissociation and conversion symptoms.

The results of comparing age and diagnoses showed that the under-20 group had a significantly higher incidence of acute stress disorder, disaster phobia, autistic spectrum disorders, and other mental disorders during childhood. The 20–64 age group had a significantly higher incidence of schizophrenia, intellectual disability, and autistic spectrum disorders. The 65-and-older group had a significantly higher incidence of dementia and sleep disorders. A comparison between age and symptoms, meanwhile, found that those under 20 had significantly more symptoms unique to children; those aged 20–64 had significantly more hallucinations and delusions, and those aged 65 and older had significantly more physical symptoms.

In Table 3, illustrating the comparison of symptoms and pre-existing mental disorders, a higher percentage of people with hallucinatory and delusional symptoms, behavioral problems, and epileptic and convulsive seizures had a history of mental disorders. In addition, we compared the post-disaster symptoms of people with a history of schizophrenia, bipolar disorder, or depression. The proportion of individuals with a history of schizophrenia was higher among those with physical symptoms, sleep problems, anxiety symptoms, symptoms related to mood and affect, hallucinatory and delusional, and behavioral problems. A higher percentage of those who had physical symptoms and symptoms related to mood and affect had a history of bipolar disorder. A higher percentage of people with sleep problems, symptoms related to mood and affect, and behavioral problems had a history of depression.

### 3.5. Diagnosis of Four Disasters and Symptoms, and Comparison with Disaster Statuses

Table 4 and Table 5 indicates the differences in disaster statuses regarding the diagnoses and symptoms of the four disasters combined.

The results of comparing diagnosis and disaster status showed that a larger per-centage of people diagnosed with PTSD were dead or missing. Among those who had injured themselves, acute stress disorder was more common (33%). Among those who had family members or acquaintances who were injured, many had adjustment disorders (28%). Among those affected by house collapses, many had depression (11%), acute stress disorder (14%), and adjustment disorder (14%). A large proportion of people with forcibly evicted from their homes many had dementia (17%) and adjustment disorder (22%). Finally, of those who lost property other than homes, a large percentage had adjustment (21%) and sleep disorders (14%). As a result of examining the symptoms and disaster status, of those who experienced a family member or other person dying or going missing, symptoms related to mood and affect (24%) and symptoms unique to children (4%) were more likely to appear. A large proportion of people who were injured many had problems sleeping (31%). Among those affected by house collapses, many had sleep problems (26%) and symptoms related to mood (18%). A large proportion of people who were forcibly evicted from their homes many had sleep problems (27%) and anxiety symptoms (30%). Finally, of those who lost property other than homes, a large percentage complained of physical symptoms (21%) and anxiety symptoms (30%).

## 4. Discussion

There are few studies similar to the current survey that investigate the characteristics of mental disease during the acute stage of disasters, categorized according to the type of disaster. We consider our study to be original as it denotes the mental and psychological characteristics in the post-disaster acute stage observed in distinct types of disasters.

### 4.1. Major Diseases and Symptoms: Acute Stage of Disasters

Over 40% of those consulted had a history of mental disease. A breakdown of the diagnoses, in addition to diseases that had previously been reported, such as acute stress disorder, adjustment disorder, and other severe stress reactions, suggests a strong need for assistance during the acute stage for patients with schizophrenia and dementia. If people encounter a disaster, their risk of being exposed to trauma increases, thus the need for treatment of PTSD, depressive disorders, and other symptoms that develop after the disaster is often anticipated. However, if people already have a serious mental illness (SMI) such as schizophrenia or bipolar disorder even before suffering the disaster, treatment for PTSD and other symptoms, together with the SMI, appears to be necessary [31]. Dementia patients lack the ability to understand and cope with stress and are at high risk of developing delusions. They are, therefore, to be considered victims requiring special attention and care in the event of a disaster [31]. Comparing the symptoms of those who had SMI before the disaster confirmed the typical symptoms of the disease. The results show that not only PTSD and anxiety disorders but also a history of SMI must be considered when various psychiatric symptoms, including mood, insomnia, and hallucinatory and delusional symptoms, are observed in the acute phase of disaster response.

Moreover, the problem is that no evidence-based knowledge of early interventions has yet been established due to the absence of reports that describe the profile of symptoms during the acute stage [15]. The overall results of our study show a high rate of incidence of anxiety symptoms, followed by sleep problems, physical symptoms, and symptoms related to mood. Therefore, when providing support in disaster-stricken areas, especially during the acute stage, the aim is to reduce victims’ anxieties and prevent incidents of sleep disorders from increasing. Regarding physical symptoms, it is said that the impact of disasters on physical and mental well-being frequently occurs together, and that worsening of physical health is likely to bring about the worsening of mental health [32]. It has already been pointed out that supporters—even if they are members of a support team specializing in psychiatry—must possess practical knowledge and skills in physical symptoms, and that their collaboration with physicians specializing in somatic medicine is essential [16].

We believe that the psychiatric symptoms that were detected in our study as well as the results of diagnoses are important for determining the types of early interventions needed during the acute stage of a disaster.

### 4.2. Risk Factors of Individuals Requiring Mental Health Support: Acute Stage of Disasters

Our findings support the disease characteristics of various mental disorders and attributes as symptom characteristics. It may be reasonable to consider that, during the acute stage of a disaster when all the victims are placed under stress, some symptoms and mental disorders that are related to the attributes of the evacuees are liable to develop. Thus far, it has been said that, because children, the elderly, and women are vulnerable to disasters and are at a greater risk than others, they require mental care [2]. The findings of our study, however, offer a different perspective, indicating the need to understand that, because of disaster stress, mental symptoms appear to be linked to the existing attributes of the evacuees, spanning all age groups and genders.

Moreover, in observing people’s disaster status, those who witnessed the death or separation of their family members, relatives, and/or acquaintances manifested acute stress disorder, posttraumatic stress disorder, symptoms related to mood and affect, and symptoms unique to children. Their injuries caused acute stress disorder, whereas insomnia and injury of family and others involved adjustment disorders. It became clear that, if their living environment changed due to their houses having collapsed or having been forcefully evacuated, numerous mental health problems were evident, such as depression, acute stress disorder, adjustment disorder, and dementia, as well as mood anxiety symptoms. Several reports have already been published on this knowledge, as factors that influence mental symptoms after a disaster [2,33]. However, our findings suggest that we may be able to predict, to a certain extent, the mental disorders and psychiatric symptoms that may occur, by taking note of the victims’ attributes and their disaster statuses from the acute stage of the disaster. For example, if there are a large number of deaths, and if the victims are young people, acute stress disorder is predicted to arise; if adults witness their house collapse, depression may occur, and if an elderly person is forced to evacuate, dementia may occur at the shelter. Ideally, those providing support should be cognizant of this knowledge and information during the acute stages of the disaster and use it when commencing mental support programs.

### 4.3. Factors That Influence Acute-Stage Mental Health: Type of Disaster

The scale of a disaster, and the scale of the damage suffered, such as the number of dead and injured, and damage to housing, as well as evacuation period, vary according to the type of disaster. Taking these into consideration, it has been reported that the morbidity rate of PTSD differs according to the type of natural disaster [34]. On confirming the population characteristics and the damage status of the affected areas in the four disasters studied on this occasion, we found that a majority of the victims of the Mt. Ontake eruption were tourists, with no evacuees once they had descended the mountain. The area affected by the Hiroshima mudslide had a high percentage of young people under the age of 15, with approximately 10% of the people who had lost their lives being minors aged under 20. Since the ratio of this age group after the Great East Japan Earthquake was reported to be approximately 5%, the high level of damage suffered by young people is a distinct characteristic [35]. Consequently, from the initial stages of support activities, DPAT joined the Hiroshima Prefectural Child Support Team and conducted various measures. The areas affected by the Kanto-Tohoku flood had a high percentage of people aged 65 years and older with as many as 60% of residents witnessing their houses collapse because before being forced to evacuate [36]. In contrast, during the Kumamoto earthquake some residents voluntarily evacuated, without suffering any damage to their homes or infrastructure, due to fear of aftershocks and anxiety about living at home amid power outages. Approximately 60% of these evacuees returned home within a week [37].

Significantly, rather than by type of disaster, the population characteristics of the affected area, the attributes of the victims and their damage situation, and the type and extent of evacuation appear to be the most influential factors of acute-stage mental health symptoms. By gathering this information, it may be possible to explain the characteristics of mental disorders as well as psychiatric and mental status during the acute stage of disasters. Therefore, when providing mental support during the acute stage of a disaster, it is deemed important, for reasons of appropriately meeting the needs for mental health, to take action by collaborating closely with the municipality’s public health nurses and mental health and welfare institutions in the affected areas in sharing the characteristics of mental health in the affected areas during normal times, as well as the content of disaster and characteristics of the evacuees, in subsequently predicting the mental disorders and symptoms that may develop. The knowledge and findings uncovered in this study are highly significant and applicable in assisting DPAT and other providers of acute-stage mental health and medical support.

### 4.4. Limitations of this Study

This study had some limitations. In addition, since DPAT mainly provide outreach support for evacuees and disaster victims, the data presented here are limited to the disaster victims who were targeted for support. Since no comparison was made for residents who did not need support, risk factors for psychiatric symptoms must be interpreted with caution. Next, choosing to study four disasters may have resulted in selection bias. Moreover, the sample size differs for each disaster, creating an imbalance in the number of people who can be analyzed, which may have weakened the statistical validity of our results. Moreover, the paucity of previous studies limits the potential for generalization. Moreover, to interpret the results in our study, we used simple tabulation, and thus, cannot rule out the possibility that apparent relationships were being shown by confounding factors. Many prefectures were still at the stage of preparing to install DPAT when the four disasters occurred, and 85% of the members of the DPAT group who were dispatched to the Kumamoto earthquake had not undergone DPAT training [38]. The accuracy of data input into DMHISS may, therefore, be questionable. There is a need to conduct analyses at a higher level of accuracy by accumulating more data and examining, in greater detail, causal relationships using longitudinal studies.

## 5. Conclusions

In investigating the data on mental health support activities after four large-scale disasters (eruptions, landslides, floods, and earthquakes), after which DPAT had conducted activities, we found that the symptoms diagnosed as posing an elevated risk during the acute stage, according to the type of disaster, were: acute stress disorder, adjustment disorder, other severe stress reactions, schizophrenia, and dementia.

Acute-stage symptoms that frequently occur after a disaster were anxiety symptoms, sleep problems, symptoms related to mood and affect, and physical symptoms.

The population characteristics of the affected area, the attributes of the victims, the degree of damage they sustained, and the characteristics of the evacuation status were the chief factors that influence acute-stage mental health symptoms.

In providing mental support during the acute stage of a disaster, not the type of disaster, population characteristics of the disaster area, attributes of the disaster victims, and the characteristics of the disaster and evacuation situation were found to influence acute mental health symptoms. It was deemed important, for reasons of appropriately meeting the needs for mental health, to take action by collaborating closely with the municipality’s public health nurses and mental health and welfare institutions in the affected areas, and sharing with them the characteristics of mental health in the affected areas during normal times, as well as characteristics of the evacuees, and subsequently being able to predict the mental disorders and symptoms that are likely to develop.

## Figures and Tables

**Table 1:** Comparison of the Basic Attributes, Diagnoses, and Symptoms of the Target Population by Disaster.

		Total (N = 1806)		Volcanic Eruption (N = 12)		Landslide (N = 86)		Flood (N = 139)		Earthquake (N = 1569)		χ2	Cramer’s V	*p*
		n	%	n	%	n	%	n	%	n	%			
Gender	Man	607	34.5	5	41.7	30	34.9	61 *	43.9	511	33.6	6.27	0.06	0.00
	Woman	1152	65.5	7	58.3	56	65.1	78	56.1	1011 *	66.4			
Age	Age under 20	149	9.5	0	0.0	29	34.5	4	3.1	116	8.6	78.19	0.16	<0.0001
	Ages 20–64	730	46.7	6	75.0	33 *	39.3	52	40.0	639	47.6			
	65 and older	685	43.8	2	25.0	22	26.2	74 *	56.9	587	43.7			
History of mental disease	Yes	799	44.2	1	8.3	33	38.4	100 *	71.9	665	42.4	74.24	0.14	<0.0001
	No	1010	55.8											
DIAGNOSIS	Dementia	96	9.2	0	0.0	1	2.0	20 *	18.5	75	8.5	15.62	0.12	0.00
	Alcoholism	30	2.9	0	0.0	0	0.0	2	1.9	28	3.2	2.43	0.05	0.49
	Schizophrenia	219	21.0	0	0.0	3	5.9	37 *	34.3	179	20.4	20.58	0.14	<0.0001
	Depression	87	8.3	0	0.0	2	3.9	8	7.4	77	8.8	2.27	0.05	0.52
	Manic depression	31	3.0	0	0.0	1	2.0	2	1.9	28	3.2	1.01	0.03	0.80
	Acute stress disorders	114	10.9	5 *	71.4	11 *	21.6	4	3.7	94	10.7	38.15	0.19	<0.0001
	PTSD	1	0.1	0	0.0	0	0.0	0	0.0	1	0.1	0.19	0.01	0.98
	Natural disaster phobia	13	1.2	0	0.0	1	2.0	0	0.0	12	1.4	1.77	0.04	0.62
	Adjustment disorder	112	10.7	1	14.3	12 *	23.5	22 *	20.4	77	8.8	22.88	0.15	<0.0001
	Other severe stress reactions	141	13.5	0	0.0	3	5.9	0	0.0	138 *	15.7	24.14	0.15	<0.0001
	Other anxiety disorders	26	2.5	0	0.0	2	3.9	1	0.9	23	2.6	1.76	0.04	0.62
	Somatoform disorder	9	0.9	0	0.0	0	0.0	2	1.9	7	0.8	1.79	0.04	0.62
	Sleep disorders	76	7.3	0	0.0	7	13.7	6	5.6	63	7.2	4.19	0.06	0.24
	Intellectual disability	27	2.6	0	0.0	1	2.0	1	0.9	25	2.8	1.68	0.04	0.64
	Autistic spectrum disorders	43	4.1	0	0.0	5 *	9.8	3	2.8	35	4.0	5.01	0.07	0.17
	Other childhood mental disorders	14	1.3	1 *	14.3	1	2.0	0	0.0	12	1.4	10.50	0.10	0.02
	Epilepsy	6	0.6	0	0.0	1	2.0	0	0.0	5	0.6	2.38	0.05	0.50
SYMPTOM	Physical symptoms	488	19.3	1	10.0	20	24.1	65 *	47.8	402	27.8	26.88	0.13	<0.0001
	Sleep problems	596	23.5	1	10.0	45 *	54.2	59 *	43.4	491	34.0	20.60	0.11	<0.0001
	Anxiety symptoms	667	26.3	8 *	80.0	23	27.7	53	39.0	583	40.4	12.04	0.09	0.01
	Symptoms related to mood and affect	419	16.5	3	30.0	32 *	38.6	29	21.3	355	24.6	9.37	0.08	0.03
	Dissociation and conversion symptoms	19	0.7	0	0.0	0	0.0	0	0.0	19	1.3	3.05	0.04	0.38
	Oppressive symptoms	17	0.7	0	0.0	0	0.0	0	0.0	17	1.2	2.72	0.04	0.44
	Hallucinations and delusions	103	4.1	0	0.0	3	3.6	13	9.6	87	6.0	4.35	0.05	0.23
	Behavioral problems	161	6.4	0	0.0	3	3.6	21 *	15.4	137	9.5	9.83	0.08	0.02
	Epileptic and convulsive seizures	8	0.3	0	0.0	0	0.0	1	0.7	7	0.5	0.64	0.02	0.89
	Drinking problems	26	1.0	0	0.0	0	0.0	5 *	3.7	21	1.5	5.57	0.06	0.14
	Disturbance of consciousness	3	0.1	0	0.0	0	0.0	0	0.0	3	0.2	0.48	0.02	0.92
	Symptoms unique to children	28	1.1	0	0.0	5 *	6.0	2	1.5	21	1.5	10.17	0.08	0.02

Note: basic attributes (gender, age, History of mental disease, diagnosis, symptoms) × disaster (volcanic eruption, landslide, Flood, earthquake) Missing data are excluded. *p* values were determined using the χ2 test. * z > 1.96, *p* < 0.05 Effect size: >0.4 (strong); 0.25–0.39 (moderate); 0.1–0.24 (weak); <0.1 (no association).

**Table 2:** Diagnosis and symptoms of Four Disasters: Comparison with the Subjects’ Basic Attributes.

		Man	Woman	χ2	φ	*p*	<20	Age 20–64	>65	χ2	Cramer’s V	*p*
		n	n				n	n	n			
DIAGNOSIS	Dementia	35	60	0.03	0.01	0.87	0	3	85 *	123.20	0.36	<0.0001
	Alcoholism	25 *	5	27.55	0.16	<0.0001	0	15	13	2.77	0.05	0.25
	Schizophrenia	104 *	114	12.04	0.11	0.00	4	158 *	43	76.66	0.28	<0.0001
	Depression	25	61	2.91	0.05	0.09	1	43	33	5.86	0.08	0.05
	Manic depression	11	20	0.06	0.01	0.81	0	16	11	2.87	0.06	0.24
	Acute stress disorders	24	87 *	13.53	0.11	<0.0001	27 *	40	37	44.02	0.21	<0.0001
	PTSD	0	1	0.60	0.02	0.62 ^a^	–	–	–	–	–	–
	Natural disaster phobia	5	8	0.00	0.00	0.95	4 *	4	4	9.35	0.10	0.01
	Adjustment disorder	43	69	0.03	0.01	0.85	10	48	52	2.24	0.05	0.33
	Other severe stress reactions	37	99 *	7.21	0.08	0.01	17	63	50	3.66	0.06	0.18
	Other anxiety disorders	9	17	0.10	0.01	0.75	0	14	11	2.46	0.05	0.29
	Somatoform disorder	1	8	2.72	0.05	0.09 ^a^	0	6	3	1.41	0.04	0.49
	Sleep disorders	16	60 *	9.57	0.10	0.00	2	21	48 *	22.64	0.15	<0.0001
	Intellectual disability	14	13	2.40	0.05	0.12	1	20 *	3	10.86	0.11	0.00
	Autistic spectrum disorders	35 *	7	39.04	0.19	<0.0001	11 *	28 *	2	32.65	0.18	<0.0001
	Other childhood mental disorders	2	11	2.77	0.05	0.08 ^a^	6 *	4	2	26.29	0.17	<0.0001
	Epilepsy	2	4	0.05	0.01	0.59	1	2	0	3.50	0.06	0.17
SYMPTOM	Physical symptoms	154	322	0.68	0.02	0.41	26	184	219 *	18.26	0.11	<0.0001
	Sleep problems	170	412 *	8.72	0.07	0.00	42	264	241	4.20	0.05	0.12
	Anxiety symptoms	161	493 *	41.53	0.16	0.00	61	290	238	3.86	0.05	0.15
	Symptoms related to mood and affect	136	274	0.12	0.01	0.73	28	192	155	5.27	0.06	0.07
	Dissociation and conversion symptoms	1	18 *	7.02	0.07	0.00 ^a^	4	10	5	4.00	0.05	0.14
	Oppressive symptoms	9	8	2.79	0.04	0.10	1	11	5	2.23	0.04	0.33
	Hallucinations and delusions	42	61	2.35	0.04	0.13	3	71 *	25	27.12	0.14	<0.0001
	Behavioral problems	91 *	67	43.98	0.16	<0.0001	16	78	57	2.27	0.04	0.32
	Epileptic and convulsive seizures	3	5	0.05	0.01	0.55 ^a^	0	4	0	4.56	0.06	0.10
	Drinking problems	21 *	5	25.96	0.13	<0.0001	0	14	8	3.79	0.05	0.15
	Disturbance of consciousness	0	3	1.54	0.03	0.30 ^a^	0	0	2	2.60	0.04	0.27
	Symptoms unique to children	15 *	13	4.94	0.06	0.03	25 *	2	0	215.63	0.38	<0.0001

Note: (diagnosis, symptoms) × basic attributes (gender, 3 age group); missing data are excluded. *p* values were determined using the χ2 test. * z > 1.96, *p* < 0.05; ^a^ Fisher’s exact test.

**Table 3:** Comparison of symptoms among pre-existing severe mental illness.

	History of Mental Illness	χ2	φ	*p*	SCZ	χ2	φ	*p*	BD	χ2	φ	*p*	MDD	χ2	φ	*p*
	n				n				n				n			
SYMPTOM																
Physical symptoms	199	2.61	0.05	0.11	25 *	20.38	0.17	<0.0001	11 *	5.11	0.09	0.02	28	2.81	0.06	0.09
Sleep problems	296	2.61	0.05	0.11	56 *	13.47	0.14	<0.0001	13	1.56	0.05	0.21	51 *	13.90	0.14	<0.0001
Anxiety symptoms	311	2.53	0.04	0.11	59 *	16.37	0.15	<0.0001	14	1.83	0.05	0.18	36	0.09	0.01	0.76
Symptoms related to mood and affect	216	3.63	0.05	0.06	27 *	29.85	0.21	<0.0001	16 *	15.16	0.15	<0.0001	45 *	24.08	0.19	<0.0001
Dissociation and conversion symptoms	10	0.14	0.01	0.71	1	1.75	0.05	0.17 ^a^	0	–	–	–	2	0.45	0.03	0.38 ^a^
Oppressive symptoms	10	3.21	0.05	0.07	3	0.01	0.00	0.59 ^a^	0	–	–	–	–	–	–	–
Hallucinations and delusions	91 *	53.05	0.20	<0.0001	77 *	169.30	0.49	<0.0001	0	–	–	–	–	–	–	–
Behavioral problems	110 *	35.78	0.17	<0.0001	43 *	6.77	0.07	0.01	2	1.30	0.04	0.20 ^a^	5 *	8.19	0.11	0.00
Epileptic and convulsive seizures	7 *	5.11	0.06	0.02 ^a^	2	0.00	0.00	0.68	0	–	–	–	1	0.01	0.00	0.62 ^a^
Drinking problems	15	3.50	0.05	0.06	3	0.58	0.03	0.33^a^	0	–	–	–	–	–	–	–
Disturbance of consciousness	2	0.09	0.01	0.62 ^a^	0	–	–	–	–	–	–	–	–	–	–	–
Symptoms unique to children	7	11.57	0.09	0.00	1	0.19	0.02	0.55 ^a^	0	–	–	–	–	–	–	–

Note: (symptoms) × (History of mental illness, serious mental illness); SCZ = schizophrenia; BD = bipolar disorder; MDD = major depressive disorder Missing data are excluded. *p* values were determined using the χ2 test. * z >1.96, *p* < 0.05; ^a^ Fisher’s exact test.

**Table 4:** Diagnosis in Four Disasters: Comparison with Disaster Statuses.

	Died or Missing				Injury of the Self				Injury of Family, Relative, Acquaintance				Collapse of House				Forced Displacement from Home				Loss of Property Other Than Home			
	n	χ2	φ	*p*	n	χ2	φ	*p*	n	χ2	φ	*p*	n	χ2	φ	*p*	n	χ2	Φ	*p*	n	χ2	φ	*p*
DIAGNOSIS																								
Dementia	1	2.53	0.05	0.08^a^	1	0.74	0.03	0.34 ^a^	1	0.29	0.02	0.45 ^a^	30	2.33	0.05	0.13	17 *	8.36	0.09	0.00	7	0.05	0.01	0.83
Alcoholism	0	–	–	–	–	–	–	–	1	0.47	0.02	0.41 ^a^	7	2.99	0.05	0.08	2	0.28	0.02	0.45 ^a^	0	–	–	–
Schizophrenia	6	1.33	0.04	0.25	2	2.36	0.05	0.12	2	1.07	0.03	0.24 ^a^	72	3.66	0.06	0.06	16	1.52	0.04	0.22	19	0.26	0.02	0.61
Depression	5	0.64	0.03	0.28 ^a^	3	0.56	0.02	0.32 ^a^	0	–	–	–	43 *	4.81	0.07	0.03	3	4.02	0.06	0.05	4	1.39	0.04	0.24
Manic depression	1	0.06	0.01	0.63 ^a^	1	0.12	0.01	0.52 ^a^	0	–	–	–	13	0.16	0.01	0.69	3	0.00	0.00	0.58	1	0.94	0.03	0.29
Acute Stress Disorders	15	26.52	0.16	<0.0001 ^a^	8 *	12.71	0.11	<0.0001	6	9.48	0.10	0.01 ^a^	56 *	6.14	0.08	0.01	11	0.01	0.00	0.95	10	0.15	0.01	0.70
PTSD	1 *	23.33	0.15	0.04 ^a^	0	–	–	–	0	–	–	–	0	–	–	–	0	–	–	–	0	–	–	–
Natural disaster phobia	0	–	–	–	1	1.71	0.04	0.26 ^a^	0	–	–	–	3	1.32	0.04	0.25	1	0.05	0.01	0.65 ^a^	2	1.03	0.03	0.27 ^a^
Adjustment Disorder	6	0.49	0.02	0.31 ^a^	2	0.15	0.01	0.52 ^a^	5 *	5.57	0.07	0.04 ^a^	56 *	7.05	0.08	0.01	22 *	15.13	0.12	<0.0001	17 *	9.33	0.09	0.00
Other severe stress reactions	2	3.00	0.05	0.08	4	0.21	0.01	0.41^a^	2	0.09	0.01	0.55 ^a^	61	1.58	0.04	0.21	9	1.82	0.04	0.18	5	4.17	0.06	0.04
Other anxiety disorders	0	–	–	–	0	–	–	–	1	0.71	0.03	0.37 ^a^	8	0.67	0.03	0.41	2	0.01	0.01	0.27 ^a^	3	0.50	0.02	0.55 ^a^
Somatoform disorder	0	–	–	–	0	–	–	–	0	–	–	–	3	0.10	0.01	0.52 ^a^	2	1.72	0.04	0.21 ^a^	1	0.13	0.01	0.52 ^a^
Sleep Disorders	3	0.01	0.00	0.62 ^a^	1	0.35	0.02	0.47 ^a^	0	–	–	–	27	0.30	0.02	0.58	9	0.54	0.02	0.46	11 *	4.98	0.07	0.03
Intellectual disability	0	–	–	–	0	–	–	–	0	–	–	–	7	1.84	0.04	0.18	1	1.08	0.03	0.26^a^	1	0.66	0.03	0.36 ^a^
Autistic spectrum disorders	1	0.36	0.02	0.46 ^a^	0	–	–	–	0	–	–	–	11	3.15	0.06	0.08	0	–	–	–	0	–	–	–
Other childhood mental disorders	1	0.33	0.02	0.45 ^a^	1	1.49	0.04	0.28 ^a^	0	–	–	–	3	1.74	0.04	0.19	0	–	–	–	0	–	–	–
Epilepsy	1	2.41	0.05	0.22 ^a^	0	–	–	–	–	–	–	–	2	0.07	0.01	0.58^a^	0	–	–	–	0	–	–	–

Note: (diagnosis) × (disaster statuses). Missing data are excluded. *p* values were determined using the χ2 test. * z > 1.96, *p* < 0.05; ^a^ Fisher’s exact test.

**Table 5:** Symptoms in Four Disasters: Comparison with Disaster Statuses.

	Died or Missing				Injury of the Self				Injury of Family, Relative, Acquaintance				Collapse of House				Forced Displacement from Home				Loss of Property Other Than Home			
	n	χ2	φ	*p*	n	χ2	φ	*p*	n	χ2	φ	*p*	n	χ2	φ	*p*	n	χ2	φ	*p*	n	χ2	φ	*p*
SYMPTOM																								
Physical symptoms	20	0.01	0.00	0.91	12	0.45	0.02	0.50	5	1.51	0.03	0.22	208	0.37	0.02	0.54	45	0.48	0.02	0.49	51 *	6.56	0.06	0.01
Sleep problems	27	0.28	0.01	0.60	19 *	5.43	0.06	0.02	11	0.31	0.01	0.58	291 *	20.57	0.11	<0.0001	68 *	10.17	0.08	0.00	57	3.86	0.05	0.05
Anxiety symptoms	31	0.60	0.02	0.44	16	0.51	0.02	0.48	14	1.64	0.03	0.20	274	0.07	0.01	0.79	75 *	10.85	0.08	0.00	73 *	14.91	0.09	<0.0001
Symptoms related to mood and affect	27 *	7.12	0.07	0.01	9	0.01	0.00	0.93	5	0.62	0.02	0.43	201 *	9.70	0.08	0.00	37	0.09	0.01	0.77	30	0.35	0.01	0.56
Dissociation and conversion symptoms	0	–	–	–	1	0.94	0.02	0.33 ^a^	1	1.61	0.03	0.27 ^a^	10	0.98	0.02	0.32	0	–	–	–	4	4.66	0.05	0.06^a^
Oppressive symptoms	0	–	–	–	0	–	–	–	0	–	–	–	11	3.82	0.05	0.05	1	0.15	0.01	0.57 ^a^	0	–	–	–
Hallucinations and delusions	3	0.44	0.02	0.36 ^a^	0	–	–	–	0	–	–	–	–	4.90	0.05	0.03	7	0.40	0.02	0.53	8	0.00	0.00	0.98
Behavioral problems	1	5.64	0.06	0.02	3	0.05	0.01	0.56 ^a^	0	–	–	–	51	7.06	0.07	0.01	11	0.63	0.02	0.43	13	0.02	0.00	0.90
Epileptic and convulsive seizures	0	–	–	–	0	–	–	–	0	–	–	–	5	1.46	0.03	0.20^a^	0	–	–	–	1	0.24	0.01	0.48 ^a^
Drinking problems	0	–	–	–	1	0.40	0.02	0.43 ^a^	1	0.83	0.02	0.35 ^a^	10	0.10	0.01	0.75	1	0.73	0.02	0.34 ^a^	1	0.58	0.02	0.38 ^a^
Disturbance of consciousness	0	–	–	–	0	–	–	–	0	–	–	–	0	–	–	–	1	2.39	0.04	0.23 ^a^	0	–	–	–
Symptoms unique to children	4*	7.25	0.07	0.03 ^a^	0	–	–	–	1	0.69	0.02	0.37 ^a^	9	1.02	0.03	0.31	2	0.07	0.01	0.57 ^a^	2	0.02	0.00	0.62 ^a^

Note: (symptoms) × (disaster statuses). Missing data are excluded. *p* values were determined using the χ2 test. * z > 1.96, *p* < 0.05; ^a^ Fisher ’s exact test.

## Data Availability

The data presented in this study are available on request from the corresponding author. The data are not publicly available due to privacy issues.
